# Regulatory mechanism of Sarmentosin and Quercetin on lipid accumulation in primary hepatocyte of GIFT tilapia (*Oreochromis niloticus*) with fatty liver

**DOI:** 10.1371/journal.pone.0309976

**Published:** 2024-09-05

**Authors:** Ruijie Guo, Kai Yu, Kai Huang, Jinghua Li, Jiao Huang, Xuhong Yang, Yaoting Wu, Dandan Wang

**Affiliations:** College of Animal Science and Technology, Guangxi University, Nanning, China; Tanta University Faculty of Agriculture, EGYPT

## Abstract

Sarmentosin (SA) and Quercetin (QC) are two active components of *Sedum Sarmentosum* Bunge, which is a traditional Chinese herbal medicine. This study aimed to investigate the role and regulatory mechanism of SA and QC in fatty liver of Genetic Improvement of Farmed Tilapia (GIFT) tilapia. GIFT tilapia were randomly divided into two groups with three replicates per treatment (30 fish in each replicate): normal diet group (average weight 3.51±0.31 g) and high-fat diet group (average weight 3.44±0.09 g). After 8 weeks feeding trial, growth index, lipid deposition, and biochemical indexes were measured. Lipid deposition, and lipid and inflammation-related gene expression were detected in a primary hepatocyte model of fatty liver of GIFT tilapia treated with SA or QC. Our results showed that high-fat diet caused lipid deposition and peroxidative damage in the liver of GIFT tilapia. The cell counting kit-8 assay results indicated that 10 μM SA and 10 μM of QC both had the least effect on hepatocyte proliferation. Moreover, both 10 μM of SA and 10 μM of QC showed lipolytic effects and inhibited the expression of lipid-related genes (*FAS*, *Leptin*, *SREBP-1c*, and *SREBP2*) in fatty liver cells. Interestingly, QC induced autophagosome-like subcellular structure and increased the expression of *IL-8* in fatty liver cells. In conclusion, this study confirmed that SA and QC improved fatty liver caused by high-fat diet, providing a novel therapeutic approach for fatty liver of GIFT tilapia.

## Introduction

Tilapia, a fast-growing and adaptable fish, is one of the world’s major freshwater farmed fish [1]. In recent years, with the development of intensive tilapia farming, non-parasitic diseases stemming from environmental factors have become increasingly prevalent and challenging [2]. Several studies have shown that high-fat feeding can cause liver steatosis, liver cirrhosis and hepatitis, resulting in fish metabolic disorder, threatening fish health and causing severe economic losses to actual production [3,4]. Therefore, alleviating diet-induced fatty liver in tilapia is of great importance. The Genetically Improved Farmed Tilapia (GIFT, *Oreochromis niloticus*) is bred from tilapia from Africa and Asia [5]. GIFT has high economic and nutritional value and currently accounts for about 75% of the total tilapia farming in China [6]. Therefore, it is urgent to screen and develop new feed additives for improving fatty liver of GIFT tilapia.

Fatty liver is one of the diseases that influence liver pathological changes and poses a significant threat to the global aquaculture [7]. In aquaculture, fatty liver can arise from multiple factors including environmental stressors, overnutrition, and genetic mutations [8]. In fish, fatty liver is linked to lipid metabolism, inflammation, and oxidative stress [9]. Numerous studies have reported the molecular pathogenesis underlying the development of fatty liver in fish. For example, melatonin mitigated lead-induced fatty liver in *Cyprinus carpio* through the gut-liver axis [10]. Copper nanoparticles elicited the development of fatty liver in *Takifugu fasciatus* through the activation of the PERK-EIF2α-SREBP-1c signaling pathway [11]. However, the mechanism of fatty liver development in GIFT tilapia are rarely reported.

*Sedum Sarmentosum* Bunge (SSB) is a traditional Chinese herbal medicine, which is a perennial herbaceous succulent plant belonging to the genus Sedum of the Crassulaceae family [12]. *Sedum Sarmentosum* Bunge extract (SSBE) has been used traditionally to treat liver inflammatory diseases in the Asian area. SSBE exerts an anti-inflammatory effect and alleviates kidney injury [13]. Sarmentosin (SA) and Quercetin (QC) are the main active ingredients in the extracts of SSB [13,14]. SA was characterized as the effective component with hepatoprotective activity by promoting Nrf2 antioxidant defense [15]. QC could reduce the excessive cholesterol accumulation *in vivo* and *in vitro* [16]. Furthermore, QC has protective effect on fatty liver degeneration induced by high-fat diet (HFD) [17,18]. However, whether SA and QC can alleviate fatty liver of tilapia caused by high fat diet remains unclear.

In recent years, the liver protective effect of traditional Chinese medicine extracts has become a hot research topic in medicine, but most of these studies are focused on mammals, and there are few studies on fish [19]. In this study, we aimed to investigate the role and regulatory mechanism of SA and QC in fatty liver of GIFT tilapia. Growth index, lipid deposition, and biochemical indexes were determined in GIFT tilapia fed with normal diet or HFD. Lipid deposition, and lipid and inflammation-related gene expression were examined in a primary hepatocyte model of fatty liver of GIFT tilapia treated with SA or QC. Our findings provide a better understanding of the function of SA and QC on lipid deposition and offer novel therapeutic strategy to alleviate fatty liver in GIFT tilapia.

## Materials and methods

### Experimental fish

All GIFT tilapia fries were obtained from the Guangxi Academy of Fishery Sciences. The extruded feed used in the experiment was purchased from Haibaolu Aquatic Feed Co., Ltd (Nanning, China), and the formula and nutritional composition of the experimental diet are shown in Table 1. The fry should be kept temporarily for 2 ~ 3 weeks before the experiment. When the fry adapts to the water environment and the weight reaches the test specification (3.5 g), the healthy and uniform fry should be selected to construct the GIFT tilapia fatty liver model. The experiment was divided into a normal diet group (ND group, basic feed) and a high-fat diet group (HFD group, additional 8% soybean oil), with 3 replicates in each group, and 30 fish in each replicate. The fry was randomly assigned to each tank (1 m×1 m×1 m) and fed twice a day (9 a.m. and 6 p.m.), and the feeding amount and the fish’s food intake were recorded. During the test, the average water temperature was 27.6 ± 0.3°C, and the dissolved oxygen content was 5.78 ± 0.26 mg/L. Use the dirt suction machine to suck out the dirt at the bottom of each test tank every day, and replace 1/3 of the test water.

**Table 1:** Nutrient level and composition of experimental diet (%).

Material	Diet groups	
	ND	HFD
Ingredient (%)		
Fish meal	5	5
Soybean meal	35	35
Rape meal	22	22
Peanut meal	10	10
Rice bran	8	8
Corn	6	6
Microcrystalline cellulose	10	0
Calcium phosphate	1.5	1.5
Choline Chloride	1	1
Vitamin premix^a^	0.5	0.5
Mineral premix^b^	0.25	0.25
Antioxidant	0.25	0.25
NaCl	0.5	0.5
Soybean oil	0	10
Total	100	100
Main nutrients		
Crude fat (%)	3.23	11.09
Crude protein (%)	35.36	35.23
Total energy (kJ/g)^c^	15.12	15.69

ND, normal diet. HFD, high-fat diet.

^a^ Vitamin premix (per kg of premix): Vitamin A 300 mg, vitamin D3 50 mg, vitamin E 1200 mg, vitamin K3 250 mg, vitamin B1 320 mg, vitamin B2 300 mg, vitamin B6 350 mg, nicotinic acid 400 mg, pantothenic acid 480 mg, folic acid 32 mg.

^b^ Mineral premix (g/kg of premix): Cu 2500 mg, Fe 75000 mg, Mn 10000 mg, Zn 15000 mg, Se 50 mg, I 2000 mg, Co 200 mg.

^c^ Total energy (kJ/g) = Total energy based on combustion values for protein, lipid, and carbohydrate.

### Sample collection

Sampling of GIFT tilapia was performed after 8 weeks of culture, and fasting for 24 h before sampling. All fish experiments were performed in accordance with the Guide for the Care and Use of Laboratory Animals of China. The Animal Protection and Use Committee of Guangxi University approved our research protocol (No.GXU-2022-256). Tricaine Methanesulfonate (MS-222) was used as an anaesthetic for tilapia, which were euthanised with MS-222. Ten tilapias in each group were anesthetized with 80 mg/L MS-222 (Redmond, WA, USA) [20], and their body weight was measured after drying the body surface water. The blood of experimental fish was collected by fishtail vein, then placed at 4°C for 4 h, centrifuged at 3000 r/min for 10 min, and the supernatant was taken and stored at—80°C for subsequent detection of serum liver function indexes, serum lipid indexes and serum antioxidant indexes.

Subsequently, the GIFT tilapia that had been blood-collected was dissected, and the liver was taken out and weighed. A portion of liver was taken into 4% paraformaldehyde solution, fixed at room temperature and used for Hematoxylin & Eosin (HE) staining. Put a part of the liver into a 4% glutaraldehyde solution, fix it at room temperature for 2 h, and then store it at 4°C for electron microscopy observation. The rest of the liver was quick-frozen with liquid nitrogen and stored at– 80°C for making frozen sections.

### Measurement of growth index

The relevant index calculation formula is as follows:

$$\text{Weight}\;\text{gain}\;\text{rate}\;\left(\text{WGR},\%\right)=\left(\text{Wt}-\text{Wo}\right)/\text{Wo}\times 100$$

$$\text{Hepatosmatic}\;\text{index}\;\left(\text{HSI},\%\right)={\text{W}}_{\text{l}}/\text{Wt}\times 100$$

$$\text{Feed}\;\text{coefficient}=\text{F}/\left(\text{Wt}-\text{Wo}\right)$$

$$\text{Specific}\;\text{growth}\;\text{rate}\;\left(\text{SGR},\%\right)=100\times \left(\text{lnWt}-\text{lnWo}\right)/\text{t}$$

Wo (g) is the initial weight of tilapia; Wt (g) is the final weight (FW) of tilapia; W_l_ (g)is the final liver weight of tilapia; t (d) is feeding days; F (g) is the amount of feed.

### HE staining

Liver tissues were fixed in 4% paraformaldehyde for 48 h and dehydrated with different concentrations of ethanol. The tissues were then embedded in paraffin and sliced to a thickness of 4 μm, after which they were baked, deparaffinised and stained with HE. The stained sections were dehydrated with pure alcohol and observed under a microscope.

### Oil red O staining

Lipid accumulation was detected by Oil red O staining as previously described [21], with some modification. First, the tilapia liver tissue was fixed in a fixative solution for 24 h, then the sucrose solution was dehydrated and then quickly frozen, embedded, and sliced (10 μm thick). Afterwards, sections were subject to Oil red O staining for 10 min, 60% isopropanol for differentiation, hematoxylin staining for 5 min, and mounting plate. Finally, observation image acquisition and analysis are carried out.

### Transmission electron microscope observation

Liver tissue structure was observed by transmission electron microscope [22]. First, the tilapia liver tissue was fixed with an electron microscope fixative and 1% osmium acid at room temperature (protected from light), dehydrated with alcohol and acetone, embedded, and sectioned. Then stain with 2% uranyl acetate saturated alcohol solution to avoid light, 2.6% lead citrate solution to avoid carbon dioxide staining, wash with ultrapure water, and dry overnight. Finally, observation image acquisition and analysis are carried out. The procedure of cell transmission electron microscope observation is the same as that of the tissue.

### Determination of serum biochemical indexes

Serum liver function indicators (alanine transaminase (ALT), aspartate transaminase (AST)), serum lipid indicators (triacylglycerol (TG), total cholesterol (T-CHO), high-density lipoprotein (HDL-C), low density lipoprotein (LDL-C)) and serum antioxidant indexes (superoxide dismutase (SOD), malondialdehyde (MDA), Glutathione peroxidase (GSH-PX)) levels were measured using commercial kits (Nanjing Jiancheng, Jiangsu, China) according to the manufacturer’s instructions. The optical density was measured with a microplate reader (BIORAD iMark). Detailed information such as the wavelength and source of the kits was shown in S1 Table.

### Primary culture of hepatocytes and treatment

Primary culture of hepatocytes was carried out based on the method of Li et al. [23]. The liver of tilapia was dissected, cut into pieces, and digested with digestive juice at 28°C until it was completely mucus. After digestion, the tissues were filtered with a 70 μm cell strainer, and centrifuged twice at 1000 rpm for 8 min. After washing twice, 20 mL of red blood cell lysate was added to the pellet and centrifuged at 800 rpm for 5 min. Afterwards, the cells were resuspended in DMEM/F12 (HAM) medium (Biological Industries, Israel) and centrifuged twice at 850 rpm for 9 min. Finally, the isolated primary hepatocytes were seeded in 96-well plates in DMEM/F12 medium containing 100 IU/mL penicillin, 100 mg/mL streptomycin and 10% fetal calf serum and cultured at 28°C with 5% CO_2_.

Sarmentosin (SA; ≥98%, HPLC) was purchased from Jinming Biotechnology Co., Ltd. (Beijing, China). Quercetin (QC; ≥98%, HPLC) was purchased from Youchun Biotechnology Co., Ltd. (Shanghai, China).

### Cell Counting Kit-8 (CCK-8) assay

According to the manufacturer’s protocol, cell viability was measured using CCK-8 (Solarbio, Beijing, China). The cell suspension of the model group was added to a 96-well plate with 100 μL per well, and incubated for 24 h in an incubator at 28°C with 5% CO_2_. SA (0, 10, 25, 50, 100 and 200 μM) or QC (0, 5, 10, 20 and 40 μM) were added to each well and the culture was continued for 24 h. Then, 10 μL of CCK-8 solution was added to each well and incubated for 1 h. Finally, the absorbance at 450 nm was measure by using an automatic microplate reader (BioTek, USA).

### Total RNA extraction and quantitative reverse transcription polymerase chain reaction (qRT-PCR)

We used Biospin Total RNA Extraction Kit (Bioer Technology, Hangzhou, USA) to extract total cellular RNA. Total RNA concentration was determined by measuring the absorbance ratio at 260 nm, and the RNA purity was determined by agarose gel electrophoresis. Then the HiScript II Q RT SuperMix for qPCR (Vazyme Biotech, Nanjing, China) was used for cDNA synthesis. The primer sequences of all genes were shown in Table 2. The primers are synthesized by TSINGKE (Guangzhou, China). The reaction mixture included 10 μL 2×SYBR Green Master Mix, 0.4 μL 10 μM Forward primer, 0.4 μL 10 μM Reverse primer, 1 μL cDNA, 8.2 μL ddH_2_O. Cycling conditions were as follows: 95°C 3 min; 95°C 10 s, 57°C 10 s, 72°C 20 s (45 cycles). Using the expression of *β-actin* as an internal control, the relative expression of mRNA of each gene was calculated according to the 2^-ΔΔCt^ method.

**Table 2:** Sequences of primers used for real-time quantitative PCR.

Target gene	Primer sequences (5’-3’)	Accession number
*SIRT1*	F: ACCAGTTCTCGTGCGTCTC	XM_005473846.4
	R: CTTCCAGTCCATTGTTGTCTCC	
*Leptin*	F: GGCATACTCGCAGACACCAA	JN688249.1
	R: ATAGGAGCACCAGACCGTAGT	
*IL-8*	F: CGCTTCAGGCTTCATCTACTT	GQ355864.1
	R: CTTCACAGGTGGCGAACAG	
*SREBP-1c*	F: ATCCTCACCGCCGACCATT	AB046200.1
	R: CAGCAGTAGACTCTCAGCCTTG	
*SREBP2*	F: TATCGGTGTAGAAGTGCTCCTC	MT134046.1
	R: CGCTGTCCATCCTATCTGTCA	
*FAS*	F: GACCTAATCACCTCCGCTCAC	GU433188.1
	R: TGCTCATATTCGCTGCTGGTT	
*CPT-1*	F: GTATCCTGCTAGTCCGTCATCC	NM_001124735.1
	R: GGTGCTCCTGTATCTTGGCTAT	
β-actin	F: CAGGGAGAAGATGACCCAGA	AY116536.1
	R: CAGGGCATAACCCTAGTAGA	

*SIRT1*, sirtuin 1; *IL-8*, interleukin 8; *SREBP-1c*, sterol regulatory element binding protein-1c; *SREBP2*, sterol regulatory element binding protein 2; *FAS*, fatty acid synthase; *CPT-1*, carnitine palmitoyltransferase1.

### Statistical analysis

Statistical analysis was performed using the Statistics Package for Social Science 22.0 software (SPSS, Inc., Chicago, IL, USA) [24]. The results are expressed as the mean ± standard deviation (SD). A t-test was utilized to assess the differences between two groups, whereas a one-way ANOVA complemented by Tukey’s post-hoc test was applied for the comparative analysis of more than two groups. A *p*-value <0.05 was deemed statistically significant.

## Results

### GIFT tilapia growth indicators

Before the breeding experiment, we weighed the initial weight of GIFT tilapia in ND the group and the HFD group, and there was no significant difference between the two groups. Compared to the ND group, HFD significantly increased the FW, WGR, and SGR of tilapia, but did not significantly influence HSI. In addition, the feed conversion ratio (FCR) of the HFD group was significantly higher than that of the ND group (Table 3).

**Table 3:** The growth performance index of GIFT tilapia in each group.

Index	Diet groups	
	ND	HFD
Initial weight (g)	3.51±0.31^a^	3.44±0.09^a^
Final weight (g)	82.37±1.31^b^	96.01±1.26^a^
Weight gain rate (%)	2246.33±85.19^b^	2694.06±78.32^a^
Specific growth rate (%)	5.26±0.05^b^	5.55±0.03^a^
Feed conversion ratio	1.22±0.01^b^	1.34±0.06^a^
Hepatosomatic index (%)	2.26±0.14^a^	2.3±0.10^a^

Data are expressed as the mean ± SD of each group of tilapia index.

^a^, ^b^ means a significant difference between groups (*P*<0.05).

### Histopathological observation of GIFT tilapia liver

**Fig 1:**
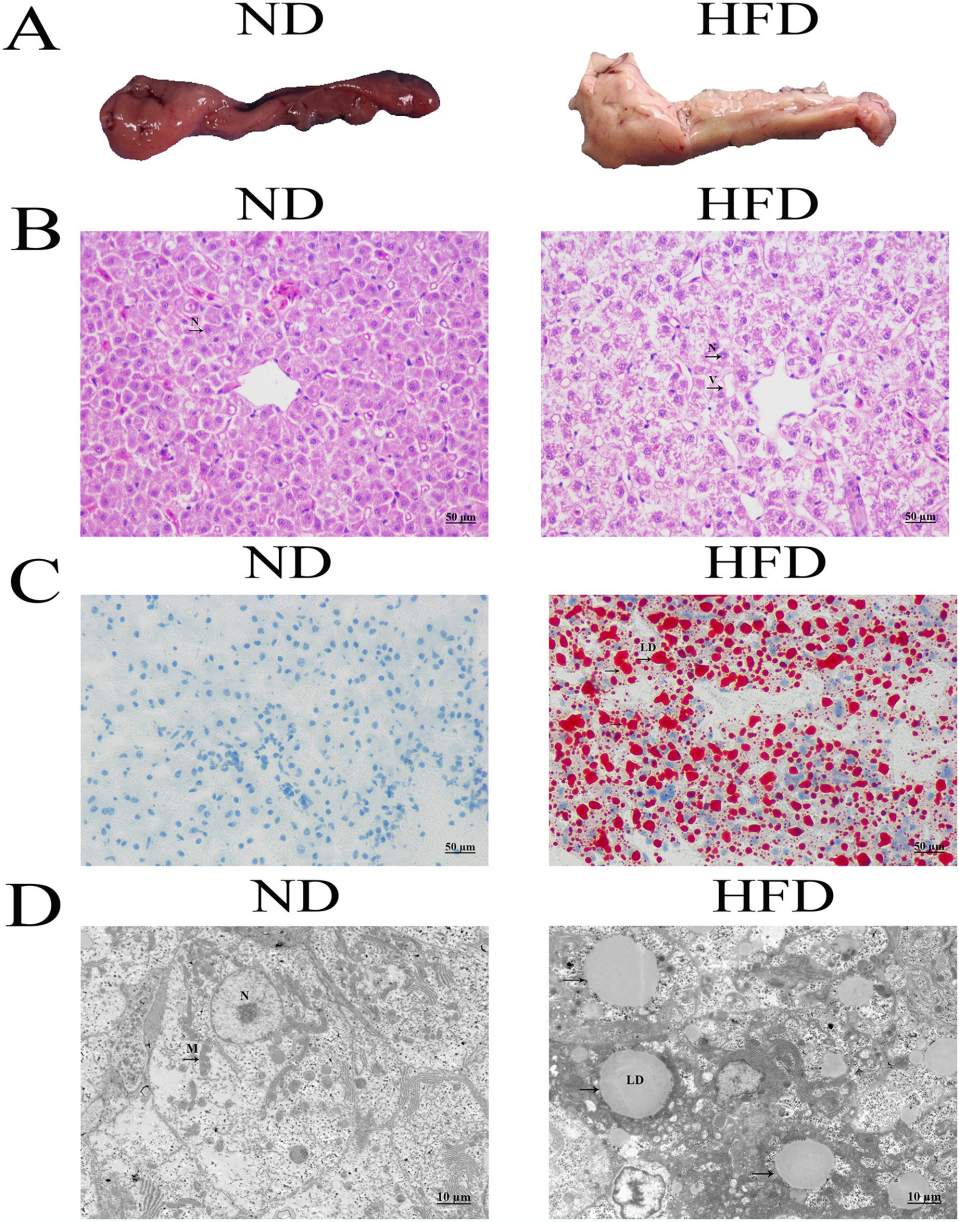
Histopathological observation of tilapia liver. (A) Representative images of liver tissues of tilapia in the ND group and the HFD group. (B) Representative images of HE staining of tilapia liver tissues in the ND group and the HFD group. (C) Representative images of Oil red O staining of tilapia liver tissues in the ND group and the HFD group. (D) Representative images of the transmission electron microscope observation results of tilapia liver tissues in the ND group and the HFD group. ND, normal diet; HFD, high-fat diet; LD, lipid drop; N, nucleus; M, mitochondria; V, vacuolation.

To investigate the effect of HFD on liver of GIFT tilapia, the liver tissue was examined. As shown in Fig 1A, the liver of GIFT tilapia fed with HFD was dark red, with a smooth surface and a relatively flat liver edge. In contrast, the liver of GIFT tilapia fed with HFD was enlarged, the liver color was white and yellow, and the edges of the tissue were not smooth. HE staining of liver tissue showed that hepatocytes in the ND group were structurally intact, with a relatively tight arrangement and a relatively uniform distribution of nuclei. Compared with the ND group, the hepatocytes in the HFD group were disorganised, with nuclei squeezed at the edge of the cells and obvious cellular vacuoles (Fig 1B). Oil Red O staining showed that the livers of GIFT tilapia fed with HFD exhibited a higher number of lipid droplets compared to those of GIFT tilapia fed with ND (Fig 1C). Moreover, the liver tissues of two groups of GIFT tilapia were observed by transmission electron microscope. As shown in Fig 1D, in the ND group, the internal structure of the liver was well-defined with distinct nuclei. The nuclear chromatin and organelles were richly populated, and the endoplasmic reticulum remained intact and well-organized. In the HFD group, the hepatocytes exhibited nuclear pyknosis and karyolysis. The cellular membranes demonstrated indistinct demarcations, and there was a noticeable depletion and disarray of organelles. A large number of lipid droplets were observed. These results indicated that HFD induced liver damage and lipid deposition in GIFT tilapia.

### GIFT tilapia serum biochemical index

**Fig 2:**
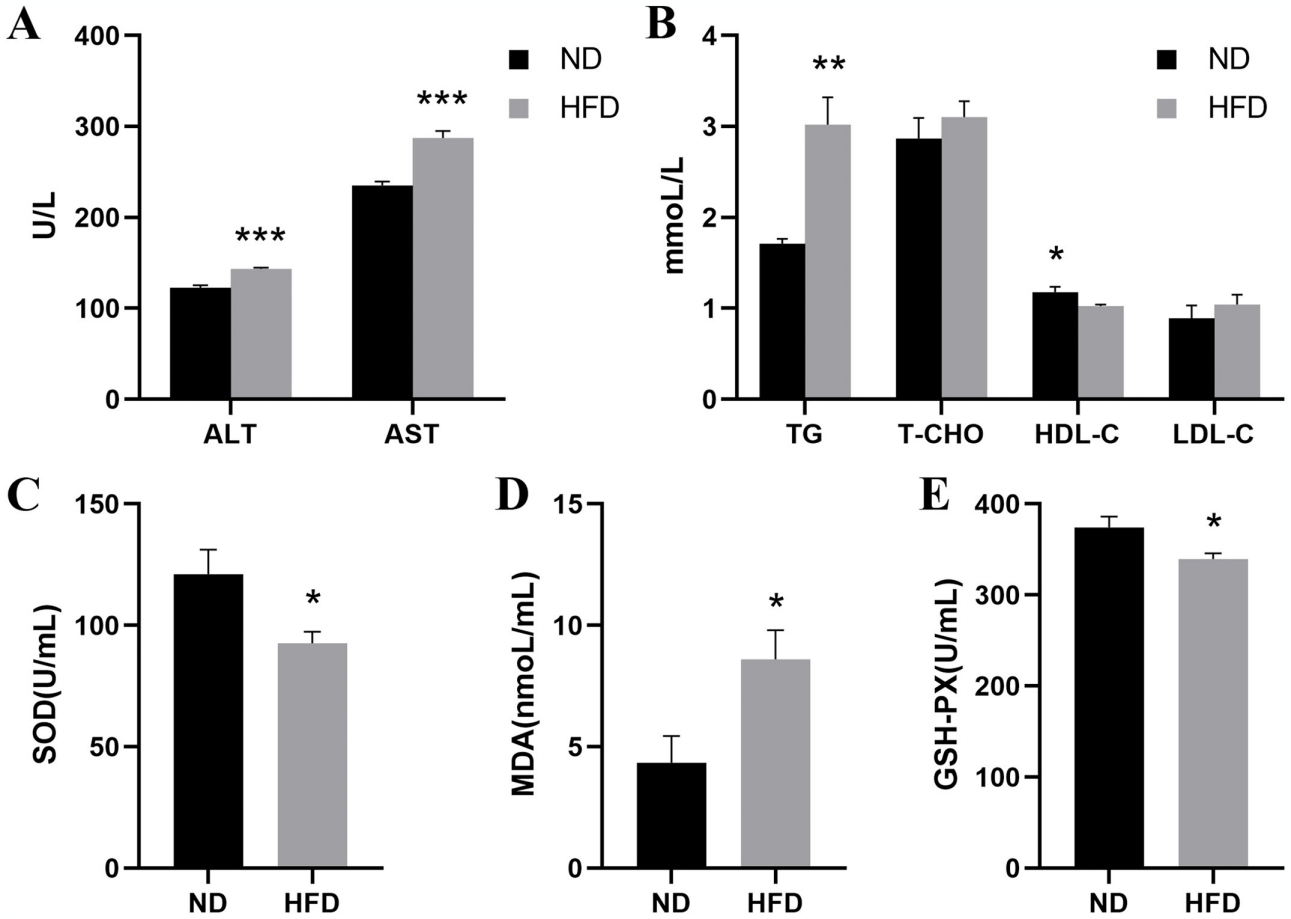
Serum biochemical indices of tilapia. (A) Serum liver function index of each group. (B) Serum lipid index of each group. (C-E) Serum antioxidant index of each group. ND, normal diet; HFD, high-fat diet; ALT, alanine transaminase; AST, aspartate transaminase; TG, triacylglycerol; T-CHO, total cholesterol; HDL-C, high-density lipoprotein; LDL-C, low density lipoprotein; SOD, superoxide dismutase; MDA, malondialdehyde, GSH-PX, glutathione peroxidase. The values are expressed as mean ± SD. The data of different groups are marked with asterisk to indicate their difference significance. *, *P* < 0.05; **, *P* < 0.01; ***, *P* < 0.001; ****, *P* < 0.0001.

After 8 weeks of high-fat feeding, the levels of ALT and AST in serum of GIFT tilapia in the HFD group were significantly higher than those in the ND group (Fig 2A). Similarly, the content of TG in the HFD group was observably higher than that in the ND group. Surprisingly, compared with the ND group, the T-CHO and LDL-C in the HFD group both showed an increasing trend, but the difference was not significant. Conversely, HDL-C in the HFD group was markedly lower than that in the ND group (Fig 2B). At the level of oxidative stress, compared with the ND group, the activities of SOD and GSH-Px in serum of the HFD group were prominently reduced, while the content of MDA in serum was significantly upregulated (Fig 2C–2E). These data suggested that HFD stimulated liver damage and oxidative damage of GIFT tilapia.

### SA and QC both reduce lipid deposition in fatty liver cells

**Fig 3:**
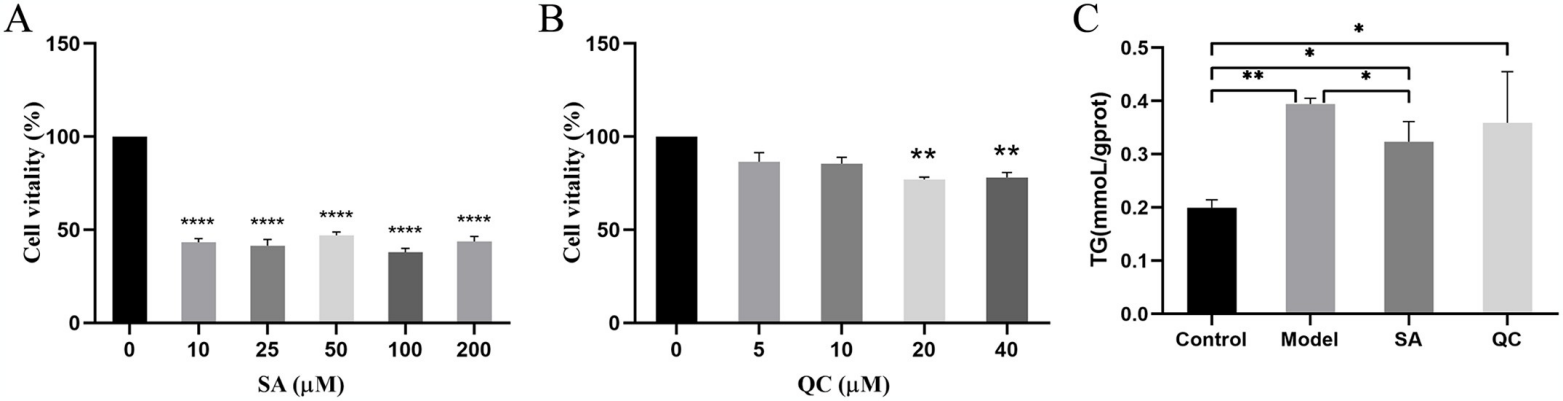
The effects of SA and QC on cell viability in the fatty liver cell model. (A, B) Effects of different concentrations of SA (A) and QC (B) on the viability of fatty liver cells were measured by the CCK-8 assay. (C) TG content in fatty liver cells. SA, sarmentosin; QC, quercetin. CCK-8, cell counting kit-8; TG, triacylglycerol. The values are expressed as mean ± SD. The data of different groups are marked with asterisk to indicate their difference significance. *, *P* < 0.05; **, *P* < 0.01; ***, *P* < 0.001; ****, *P* < 0.0001.

To investigate the effect of SA and QC on fatty liver, we constructed primary hepatocyte model of fatty liver of GIFT tilapia and treated with SA and QC. When the SA concentration was from 10 μM to 200 μM, the cell viability changed smoothly, and the cell concentration corresponding to 10 μM was close to 50%, which was in line with CC50 (Concentration of Cytotoxicity 50%) (Fig 3A). Thus, 10μM of SA was used in the subsequent *in vitro* experiment. Furthermore, when the concentration of QC was no more than 10 μM, there was no significant effect on hepatocyte viability, so we choose 10μM of QC for subsequent experiments (Fig 3B). We isolated primary hepatocytes from GIFT tilapia of the ND group and HFD group and incubated the hepatocytes of a model group with SA and QC, respectively. The results showed that only SA significantly reduced the content of TG (Fig 3C).

**Fig 4:**
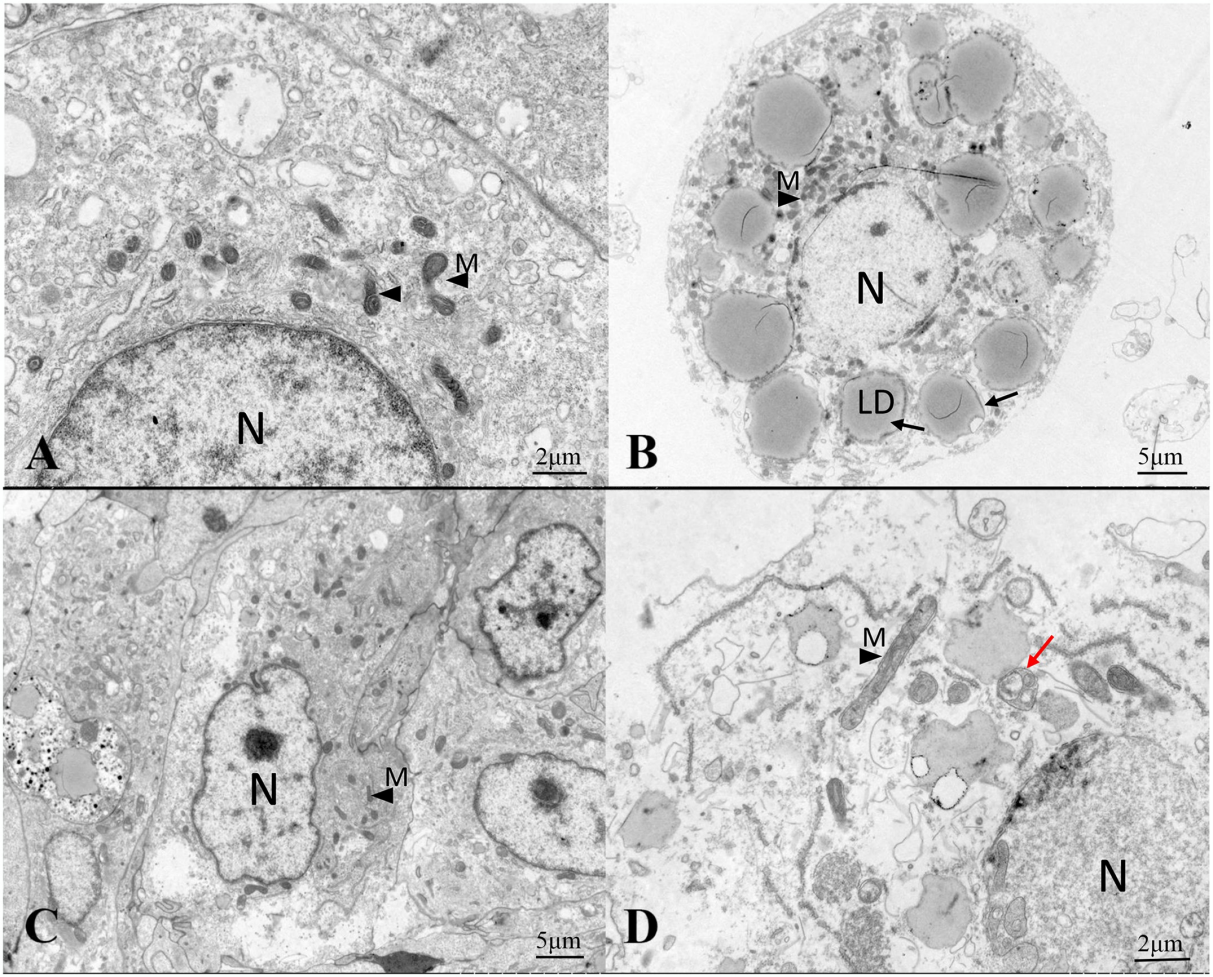
Transmission electron microscope observation in primary hepatocytes. (A) Transmission electron microscope observation of control primary hepatocytes. (B) Transmission electron microscope observation of fatty liver cells. (C) Transmission electron microscope observation of fatty liver cells treated with SA. (D) Transmission electron microscope observation of fatty liver cells treated with QC. Red arrowhead pointed to autophagosome-like subcellular structure. SA, sarmentosin; QC, quercetin; LD, lipid drop; N, nucleus; M, mitochondria.

To further observe the effects of SA and QC on hepatocytes of the model group, transmission electron microscopy was used for observation. As shown in Fig 4A, the control group displayed an absence of prominent lipid droplets, with intact cell structure and no discernible damage to the cell membrane. Conversely, the model group exhibited a substantial accumulation of lipid droplets. The mitochondria appeared swollen with extensive vacuolation observed within their interior (Fig 4B), indicating that an in vitro model of fatty liver cells has been successfully established. Compared to the model group, the SA group and QC group showed a significant reduction in lipid droplets, with the cell structure remaining intact (Fig 4C and 4D). Importantly, the autophagosome-like subcellular structure appeared in the QC group (Fig 4D), which suggested that QC may activate cell autophagy. Overall, our results demonstrated that SA and QC reduced lipid deposition caused by HFD.

### Effects of SA and QC on lipid and inflammation-related genes in fatty liver cells

**Fig 5:**
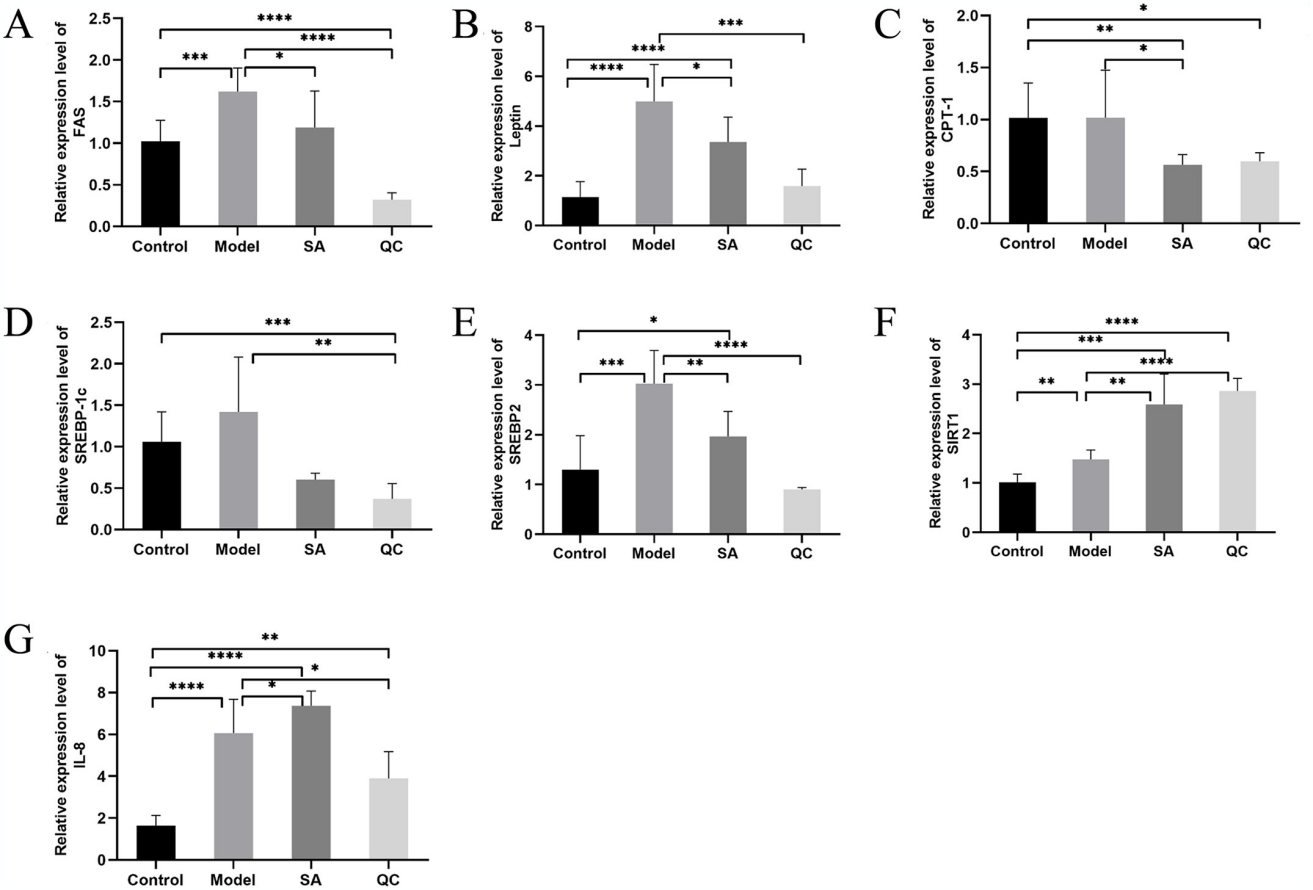
Effects of SA and QC on the expression of lipid and inflammation-related genes in the fatty liver cell model. Expression of *FAS* (A), *Leptin* (B), *CPT-1* (C), *SREBP-1c* (D), *SREBP2* (E), *SIRT1* (F), and *IL-8* (G) was detected by qRT-PCR. SA, sarmentosin; QC, quercetin; *SIRT1*, sirtuin 1; *IL-8*, interleukin 8; *SREBP-1c*, sterol regulatory element binding protein-1c; *SREBP2*, sterol regulatory element binding protein 2; *FAS*, fatty acid synthase; *CPT-1*, carnitine palmitoyltransferase1. The values are expressed as mean ± SD. The data of different groups are marked with asterisk to indicate their difference significance. *, *P* < 0.05; **, *P* < 0.01; ***, *P* < 0.001; ****, *P* < 0.0001.

To explore the mechanism of SA and QC on lipid deposition regulation, we conducted qRT-PCR detection of lipid and inflammation-related genes. In addition to *CPT-1*, compared with the control group, the model group significantly increased the expression of lipid-related genes such as *FAS*, *Leptin*, *SREBP-1c*, and *SREBP2*, while SA and QC treatment weakened the expression of these genes (Fig 5A–5E). At the same time, we also detected the SIRT1 expression and found that SA and QC remarkably increased SIRT1 expression in fatty liver cells (Fig 5F). Moreover, SA obviously increased the expression of IL-8 in fatty liver cells, while the result of the QC group was opposite to SA (Fig 5G). Thus, our results confirmed that SA and QC might exert lipid-lowering effects via regulating lipid-related genes.

## Discussion

Fatty liver caused by HFD occurs all over the world, whose main pathological features include hepatic lipid accumulation and steatosis. At present, the excessive addition of fat in aquatic feed often leads to fatty liver in fish, which affects the growth and development of fish [25]. In view of this, it is urgent to find drugs that can improve fish fatty liver. Here, we found that compared with GIFT tilapia fed with ND, the liver of GIFT tilapia in the HFD group had obvious fat deposition and oxidative damage. Moreover, SA and QC could alleviate the lipid deposition and inhibited the expression of lipid-related genes in fatty liver cells. Our research provided a reliable scientific basis for the development of new drugs to treat fatty liver in GIFT tilapia.

HFD has been reported to induce fatty liver in fish [26]. In this study, we investigated whether HFD could induce fatty liver in GIFT tilapia. Histopathological observation of GIFT tilapia liver showed that in the HFD group, the liver gradually turned white, the volume became larger, and there were obvious vacuoles between cells. Simultaneously, the liver tissue in the HFD group gathered lipid droplets, the tissue structure was disrupted, the mitochondria were swollen and the endoplasmic reticulum was abnormal. These indicators and symptoms are in line with the NAFLD model scoring system (NAS) for the determination of fatty liver, such as the liver tissue morphology, the degree of liver degeneration, and the degree of vacuolation [27]. Moreover, the HDL-C and TG indicators in the serum are also used as the criteria for fatty liver. Reduced HDL-C causes coronary heart disease, hypertriglyceridemia, and liver dysfunction [28]. High content of TG is easy to cause fatty liver, coronary heart disease, and atherosclerosis [29]. In accordance with these studies, after high-fat feeding GIFT tilapia, the content of TG was significantly increased, while HDL-C was decreased. These data suggested that HFD caused fatty liver in GIFT tilapia.

AST and ALT are powerful indicators to reflect the level of liver injury [30]. Studies have shown that patients with NAFLD and NASH had higher levels of AST and ALT in the serum [31]. Following the administration of HFD to yellow catfish, the levels of ALT and AST were elevated compared to those in the normal group [32]. Similarly, our results showed that the contents of AST and ALT in the HFD group are significantly higher than that in the ND group, indicating that long-term high-fat feeding can lead to liver dysfunction of GIFT tilapia. In addition, HFD can lead to excessive free fatty acids in the blood circulation and systemic inflammation [33]. Therefore, SOD, GSH-PX, and MDA are important standards for testing whether the body is healthy. Research has shown that the increase of MDA content was accompanied by oxidative stress and liver injury [34]. The activity of GSH-PX and T-SOD was weakened, which promoted apoptosis and liver steatosis [35]. In our study, the content of MDA was significantly higher, whereas the activity of SOD and GSH-Px in the HFD group was observably lower than in the ND group. This data suggested that HFD could induce oxidative stress and liver damage in tilapia, making it particularly important to find drugs that effectively address fatty liver disease.

Chinese medicine is increasingly being used in the treatment of fatty liver because of its unique advantages such as multi-target and multi-channel mechanism [36]. SSB, a Chinese medicine, has a protective effect on liver injury, but the specific therapeutic mechanism remains unclear. In this study, we found that compared with the control group, hepatocyte in the model group were filled with lipid droplets and mitochondria were swollen, while both SA and QC treatment significantly reduced lipid droplets. Interestingly, autophagosomes were observed in the QC group. Allaire et al. found that autophagy could improve liver damage and play an important role in the occurrence and development of NAFLD [37]. Las et al. reported that a high concentration of lipids will prevent the fusion of lysosomes and autophagosomes, thereby reducing the activity of lysosomal acidification/hydrolase to inhibit autophagy [38]. Therefore, QC and SA could reduce lipid deposition in fatty liver cells of GIFT tilapia.

Abnormal lipid metabolism is generally associated with high expression of fat synthesis genes. In the study, compared with the control group, the expression of *FAS*, *Leptin*, *SREBP-1c* and *SREBP2* in the model group was significantly increased, while SA and QC could alleviate such abnormal expression to a certain extent. Similarly, Ramgopal et al. discovered that that *Terminalia paniculata* bark extract can treat fatty liver in rats by reducing the expression of *FAS* mRNA in the liver [39]. *SREBP* is a major participant in lipid metabolism, controlling the body’s synthesis and uptake of lipids [40]. Chen et al. found that Chlorella extract significantly down-regulated the expression level of *SREBP-1c*, improved lipid metabolism and reduced fat accumulation in hyperlipidemia rats [41]. Reduction of SREBP2 inhibited serum lipid levels [42]. Additionally, *SIRT1* exerts a favorable influence in the regulation of hepatic lipid metabolism, and the stimulation of *SIRT1* activity impedes the advancement of fatty liver diseases [43]. Guo et al. found that *SIRT1* could reduce the protein expression of *SREBP-1c* and its downstream targets (*FAS*) [44]. Correspondingly, in this study, the expression of *SIRT1* mRNA in the SA group and the QC group was higher than that in the model group. Therefore, SA and QC might increase the lipid metabolism rate of GIFT tilapia high-fat hepatocyte and reduce lipid accumulation through the *SIRT1/SREBP-1c*/*FAS* pathway.

Fatty liver is often accompanied by inflammation. *IL-8* is an inflammatory cytokine, which is highly expressed in fatty liver diseases, promotes inflammation and aggravates liver injury [45]. QC is an active substance with anti-inflammatory effect, which blocks the activation of MAPK and NF-κB signaling pathway to reduce the expression of inflammatory mediators such as *IL-8* [46]. In our study, HFD resulted in a significant increase in the expression of IL-8 mRNA in hepatocytes. However, the expression of *IL-8* was weakened in denatured hepatocytes treated with QC. Therefore, these data suggested that QC might reduce fatty liver symptoms caused by HFD via inhibiting inflammation.

## Conclusion

In conclusion, our study reveals that HFD induced lipid deposition, liver damage and oxidative damage of GIFT tilapia. Interestingly, SA and QC reduced the accumulation of lipid and decreased the expression of lipid-related genes in GIFT tilapia with fatty liver. SA and QC has the potential to prevent fatty liver in GIFT tilapia.

## Supporting information

S1 Table The detailed information for the detection of antioxidant capabilities (SOD, CAT, GSH-PX) and the serum biochemical indexes. (DOCX)

S1 Raw images (PDF)

S2 Raw images (PDF)

## Abbreviations

ALT: alanine transaminase
ANOVA: one-way analysis of variance
AST: aspartate transaminase
CCK-8: cell counting kit-8
*CPT-1*: carnitine palmitoyltransferase1
FAS: fatty acid synthase
FCR: feed conversion ratio
GIFT: Genetic Improvement of Farmed Tilapia
GSH-PX: glutathione peroxidase
HDL-C: high-density lipoprotein
HFD: high-fat diet
HSI: hepatosmatic index
*IL-8*: interleukin 8
LDL-C: low density lipoprotein
MDA: malondialdehyde
NAFLD: non-alcoholic fatty liver disease
ND: normal diet
QC: quercetin
qRT-PCR: quantitative reverse transcription polymerase chain reaction
SA: sarmentosin
SD: standard deviation
SGR: specific growth rate
*SIRT1*: sirtuin 1
SOD: superoxide dismutase
*SREBP-1c*: sterol regulatory element binding protein-1c
*SREBP2*: sterol regulatory element binding protein 2
SSB: *Sedum Sarmentosum* Bunge
SSBE: *Sedum Sarmentosum* Bunge extract
T-CHO: total cholesterol
TG: triacylglycerol
WGR: weight gain rate
