# Supplementary material for: Regulatory mechanism of Sarmentosin and Quercetin on lipid accumulation in primary hepatocyte of GIFT tilapia (Oreochromis niloticus) with fatty liver
Source: PLoS One. 2024 Sep 5;19(9):e0309976. doi: 10.1371/journal.pone.0309976 (PMC11376590; doi:10.1371/journal.pone.0309976)
Supplement: S1 Table — (DOCX) [file pone.0309976.s001.docx]

| Name | Manufacturer | Cat No. | wavelength |
| --- | --- | --- | --- |
| ALT | Nanjing Jiancheng | C009-2-1 | 510 nm |
| AST | Nanjing Jiancheng | C010-2-1 | 510 nm |
| T-CHO | Nanjing Jiancheng | A111-2-1 | 500 nm |
| TG | Nanjing Jiancheng | A110-1-1 | 500 nm |
| LDL-C | Nanjing Jiancheng | A113-1-1 | 600 nm |
| HDL-C | Nanjing Jiancheng | A112-1-1 | 600 nm |
| SOD | Nanjing Jiancheng | A001-3-2 | 450 nm |
| MDA | Nanjing Jiancheng | A003-1-2 | 532 nm |
| GSH-PX | Nanjing Jiancheng | A005-1-2 | 412 nm |

**Table S1 The detailed information for the detection of antioxidant capabilities (SOD, CAT, GSH-PX) and the serum biochemical indexes.**
