# Supplementary figures and images for: Regulatory mechanism of Sarmentosin and Quercetin on lipid accumulation in primary hepatocyte of GIFT tilapia (Oreochromis niloticus) with fatty liver
Source: PLoS One. 2024 Sep 5;19(9):e0309976. doi: 10.1371/journal.pone.0309976 (PMC11376590; doi:10.1371/journal.pone.0309976)

**Fig.1A ND**

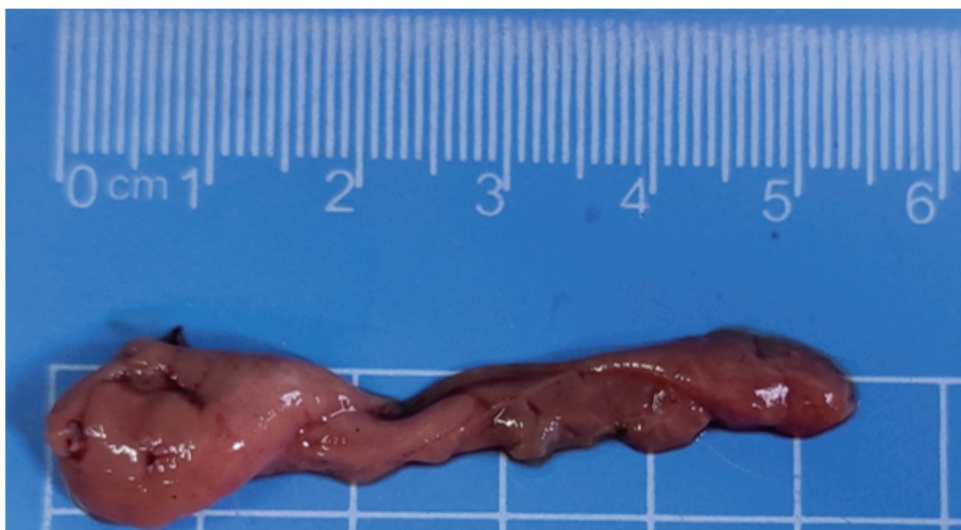

**Fig.1A HFD**

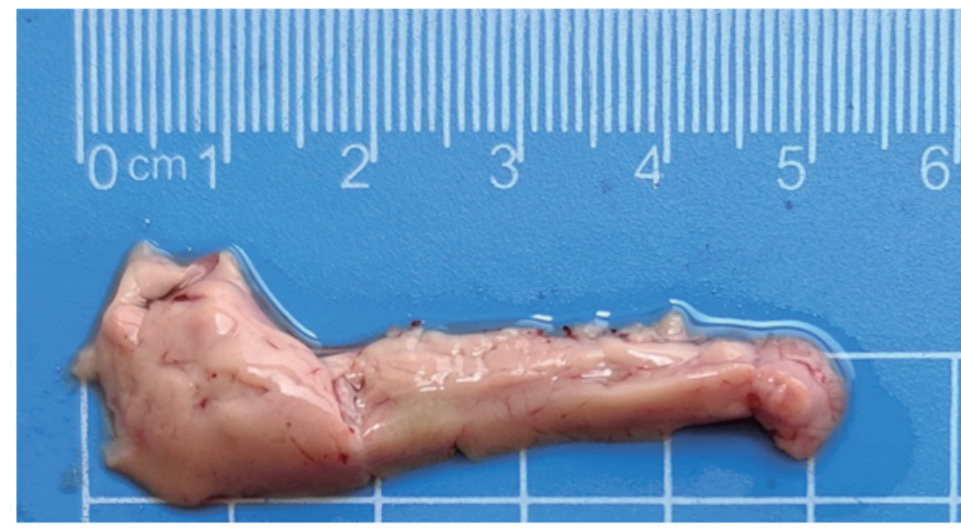

**Fig.1B ND**

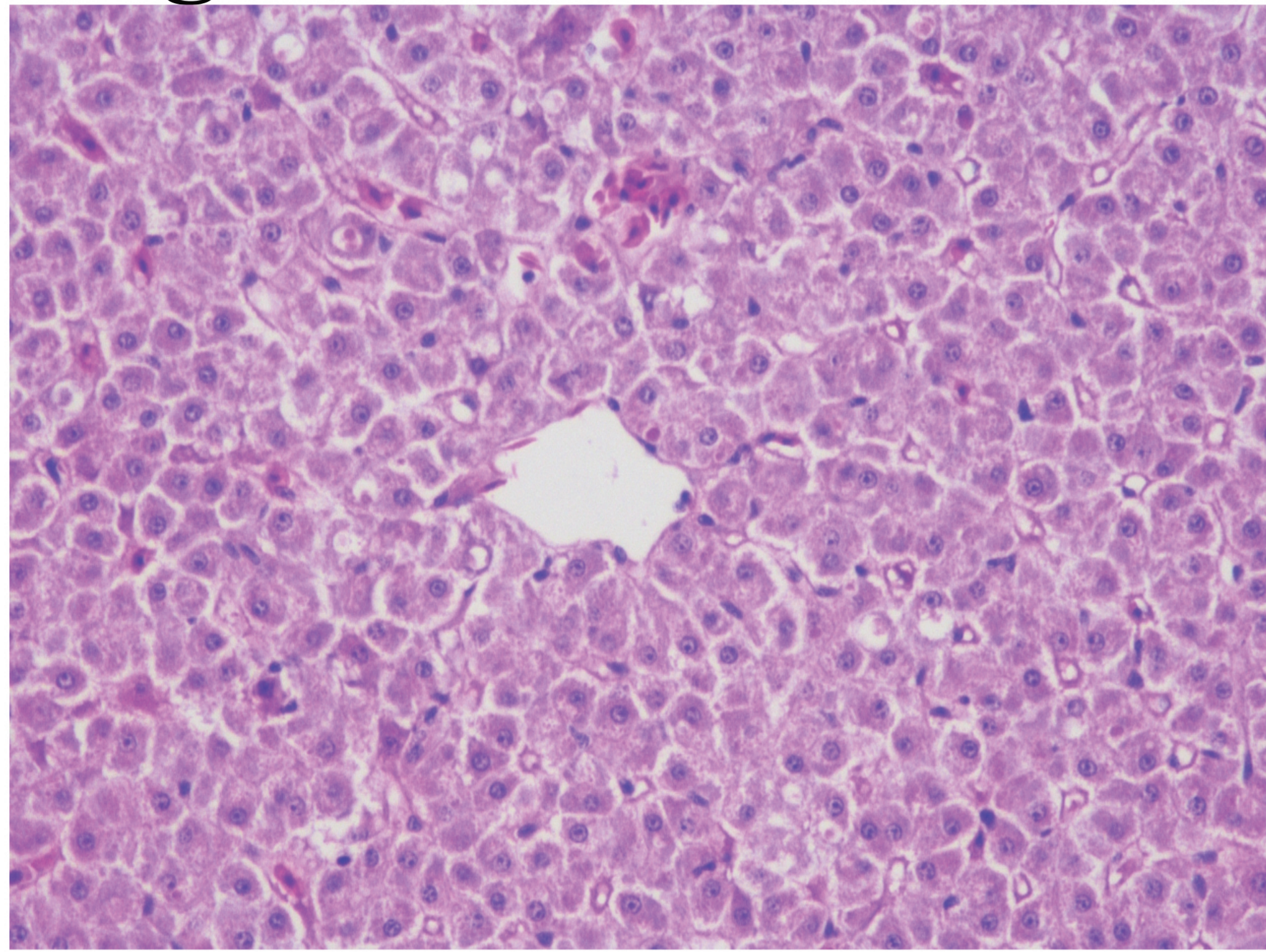

**Fig.1B HFD**

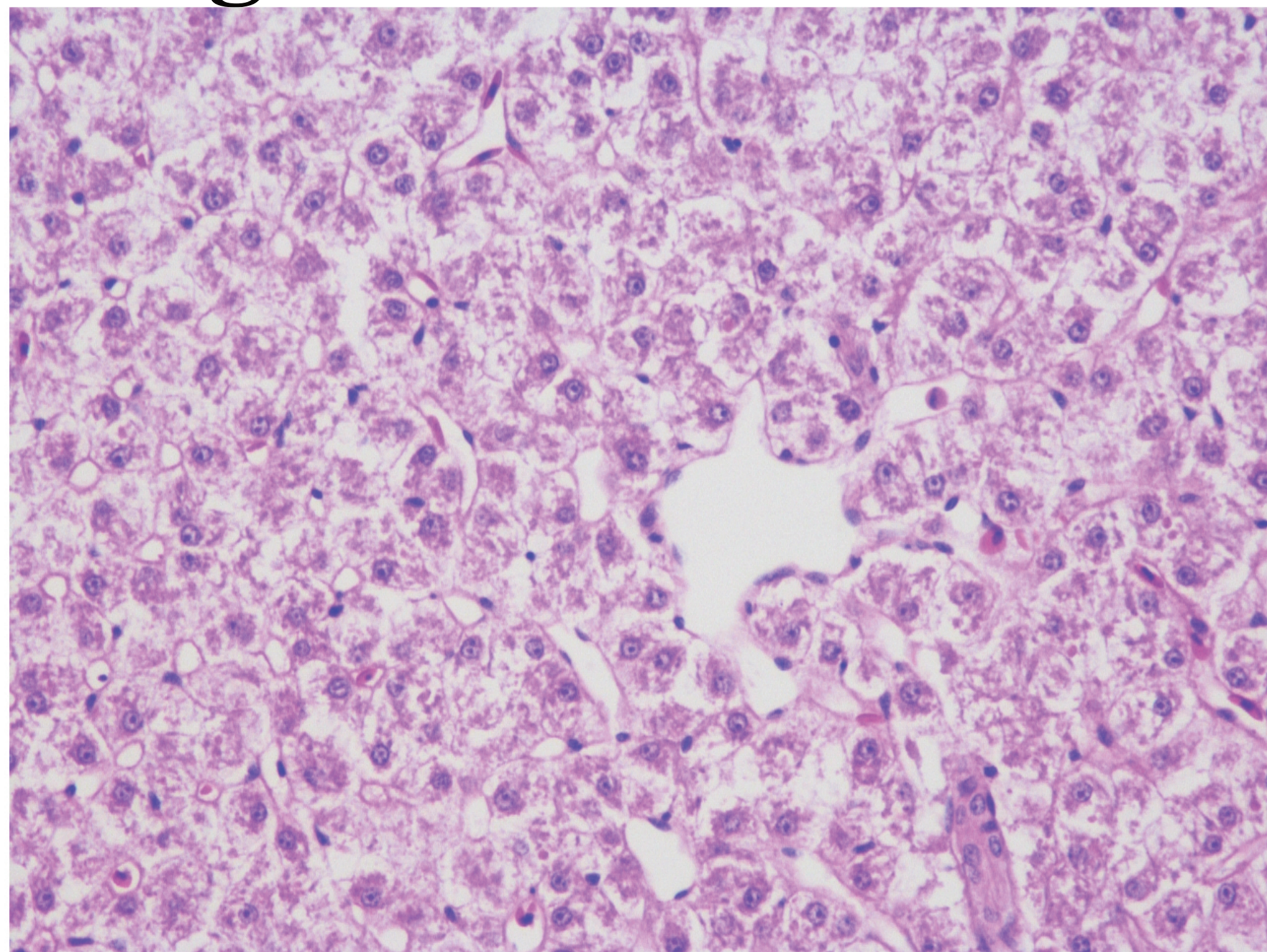

**Fig.1C ND**

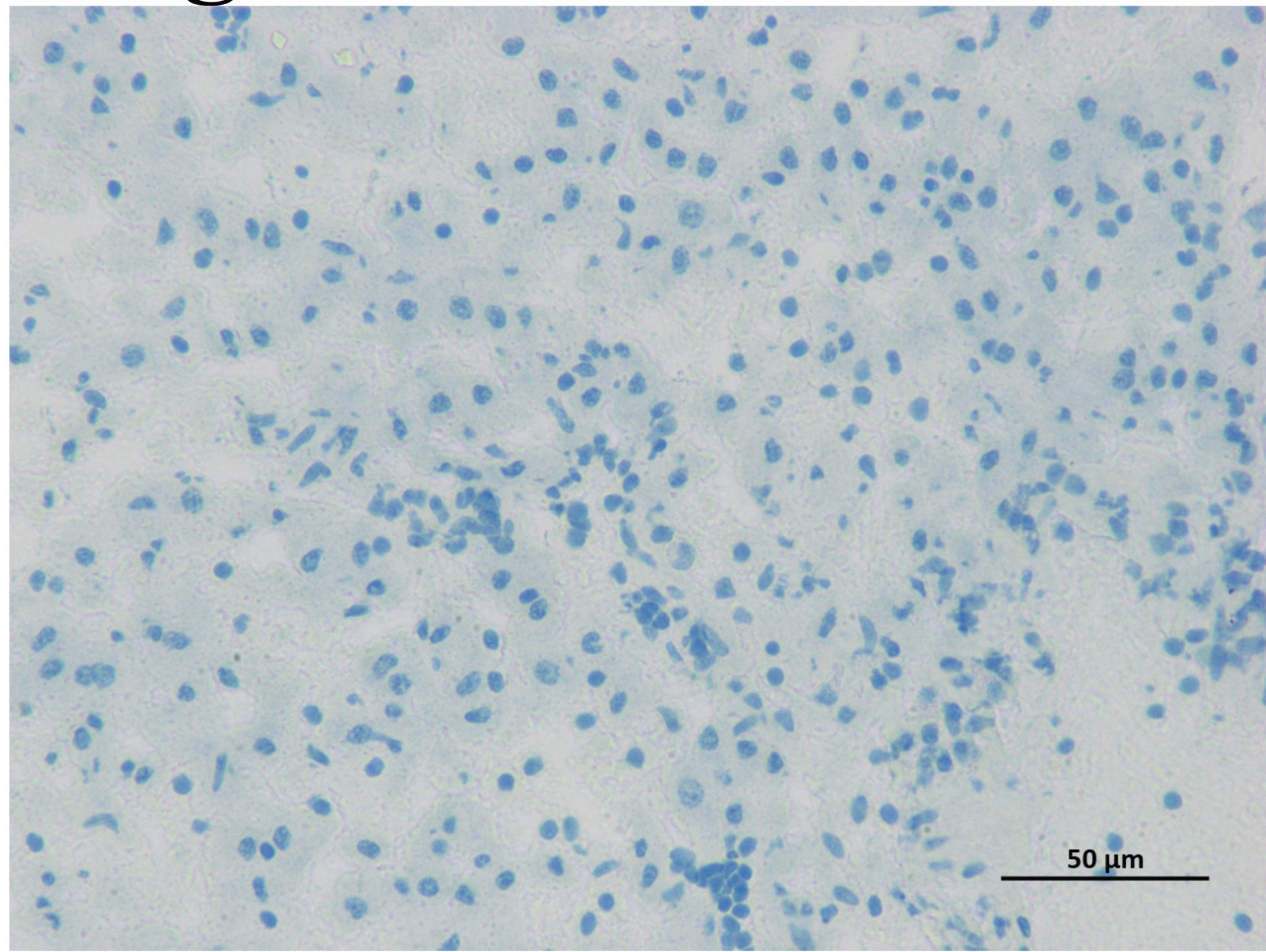

**Fig.1C HFD**

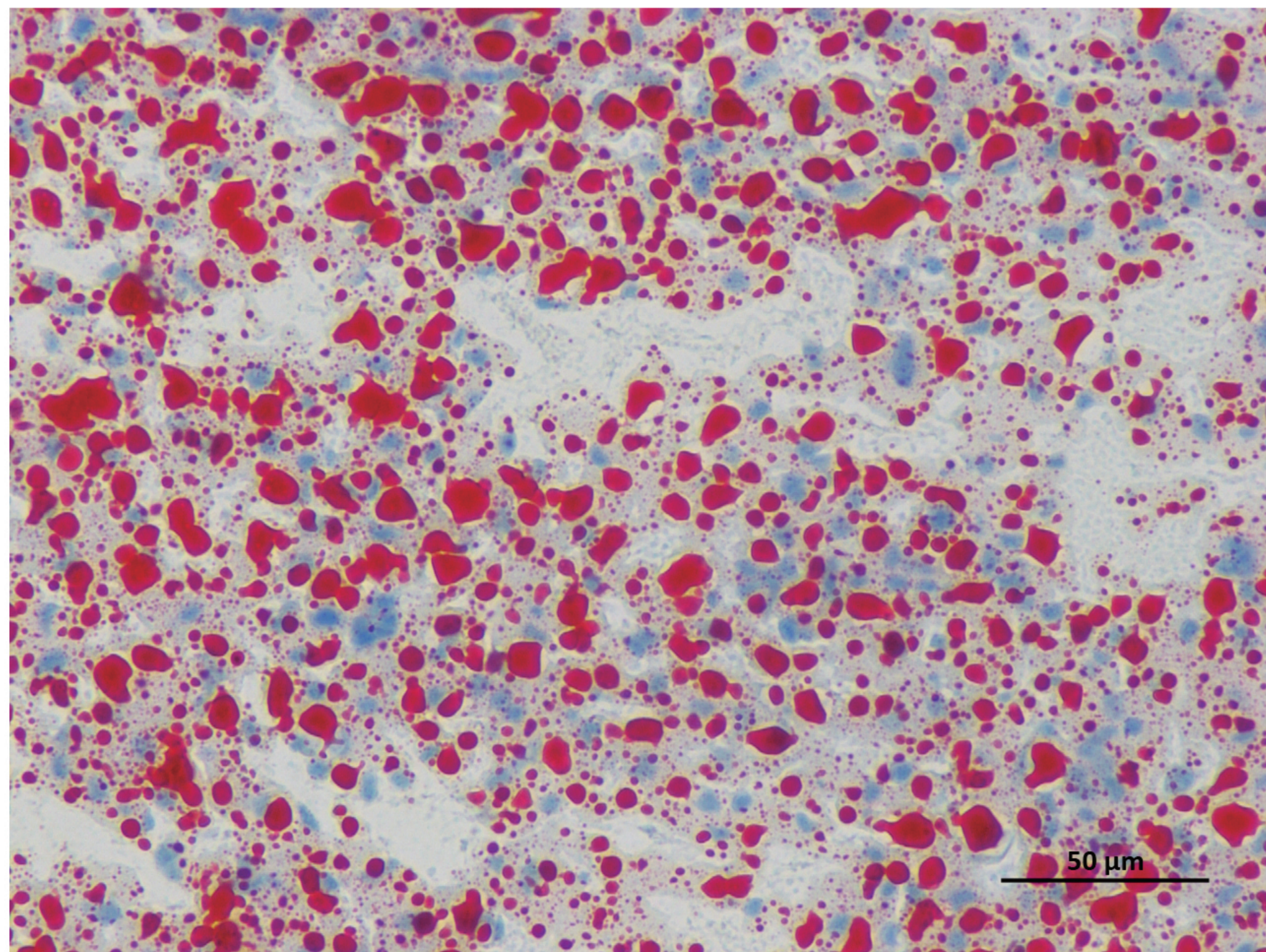

**Fig.1D ND**

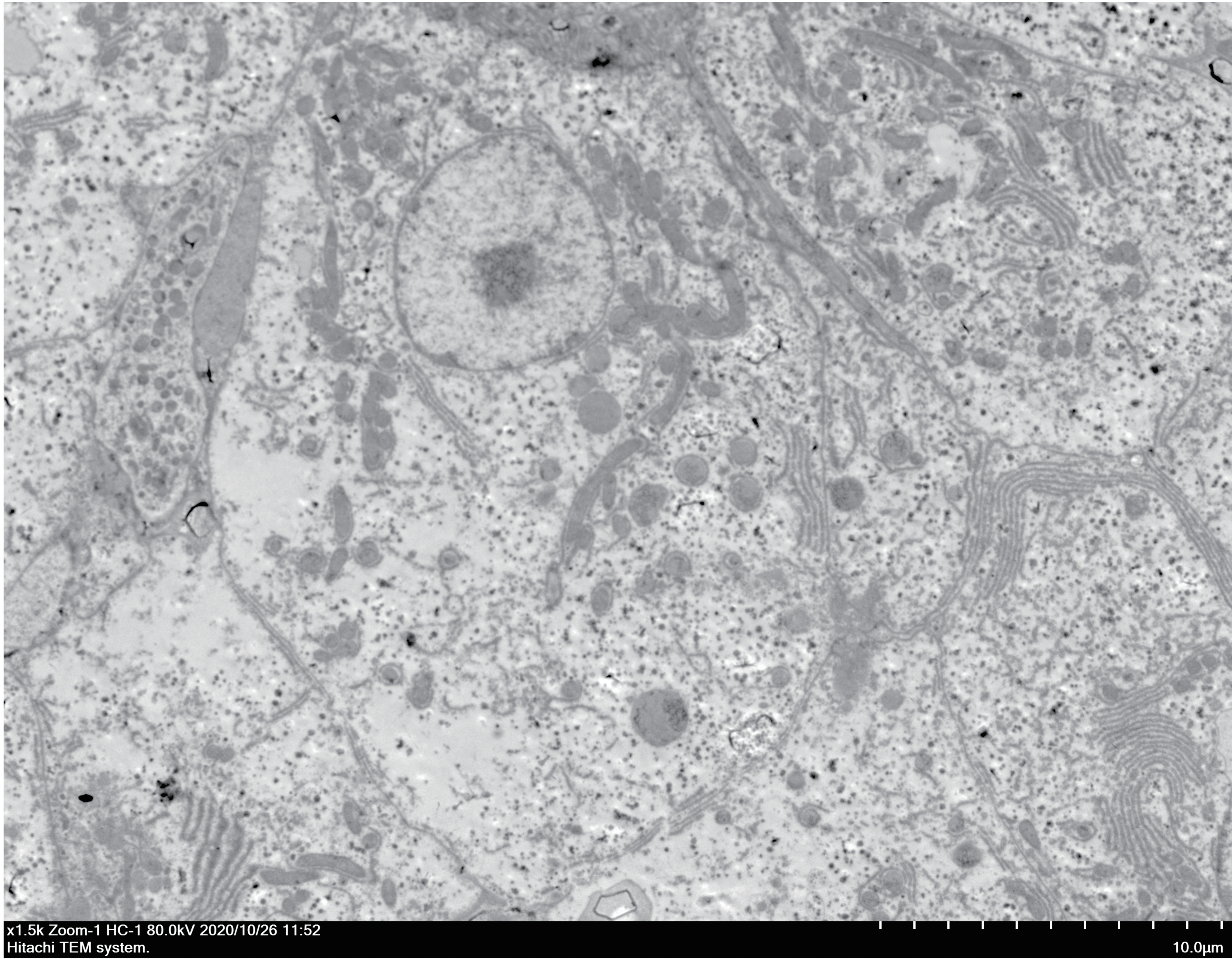

**Fig.1D HFD**

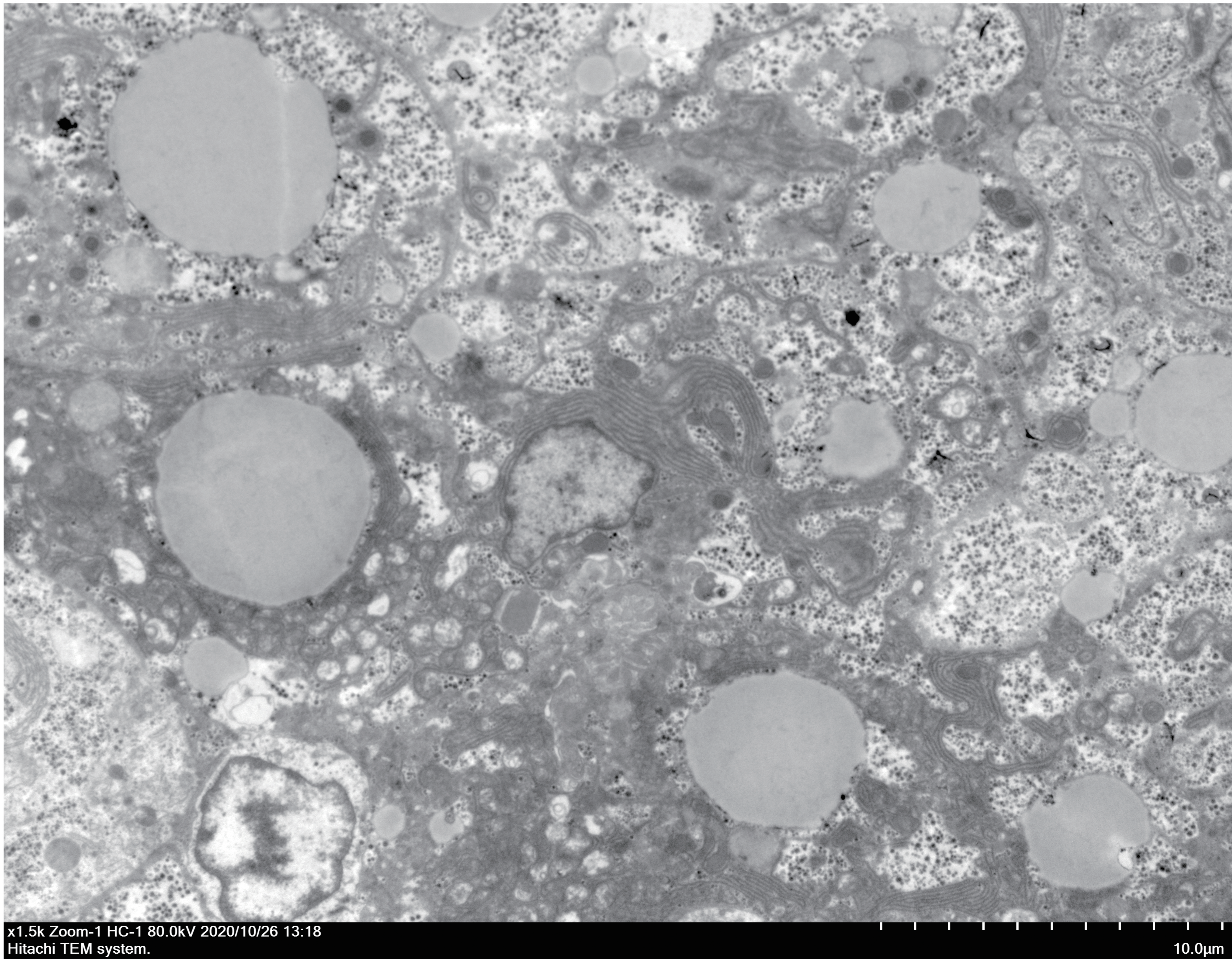

Supplement: S1 Raw images — (PDF) [file pone.0309976.s002.pdf]

**Fig.4A**

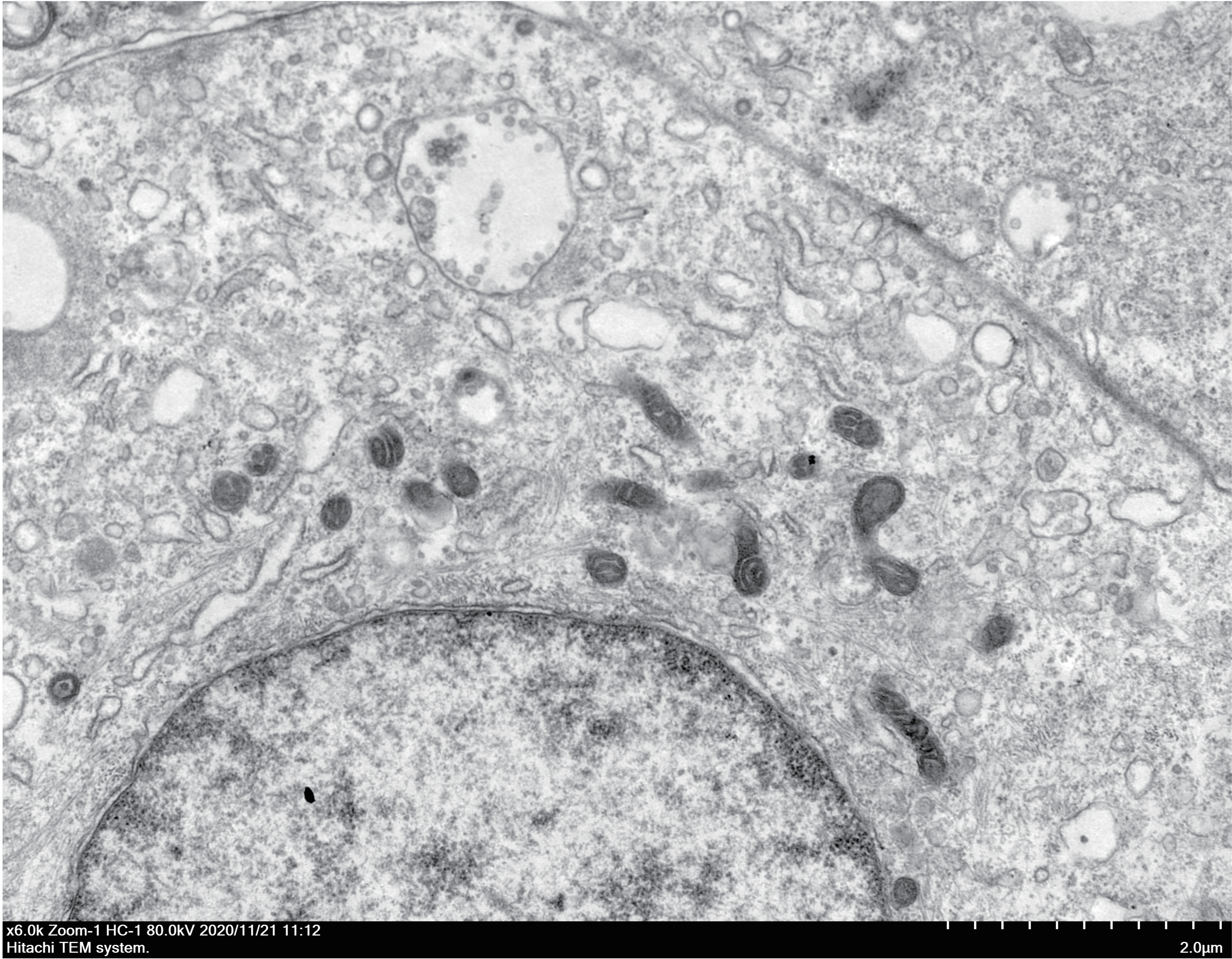

**Fig.4B**

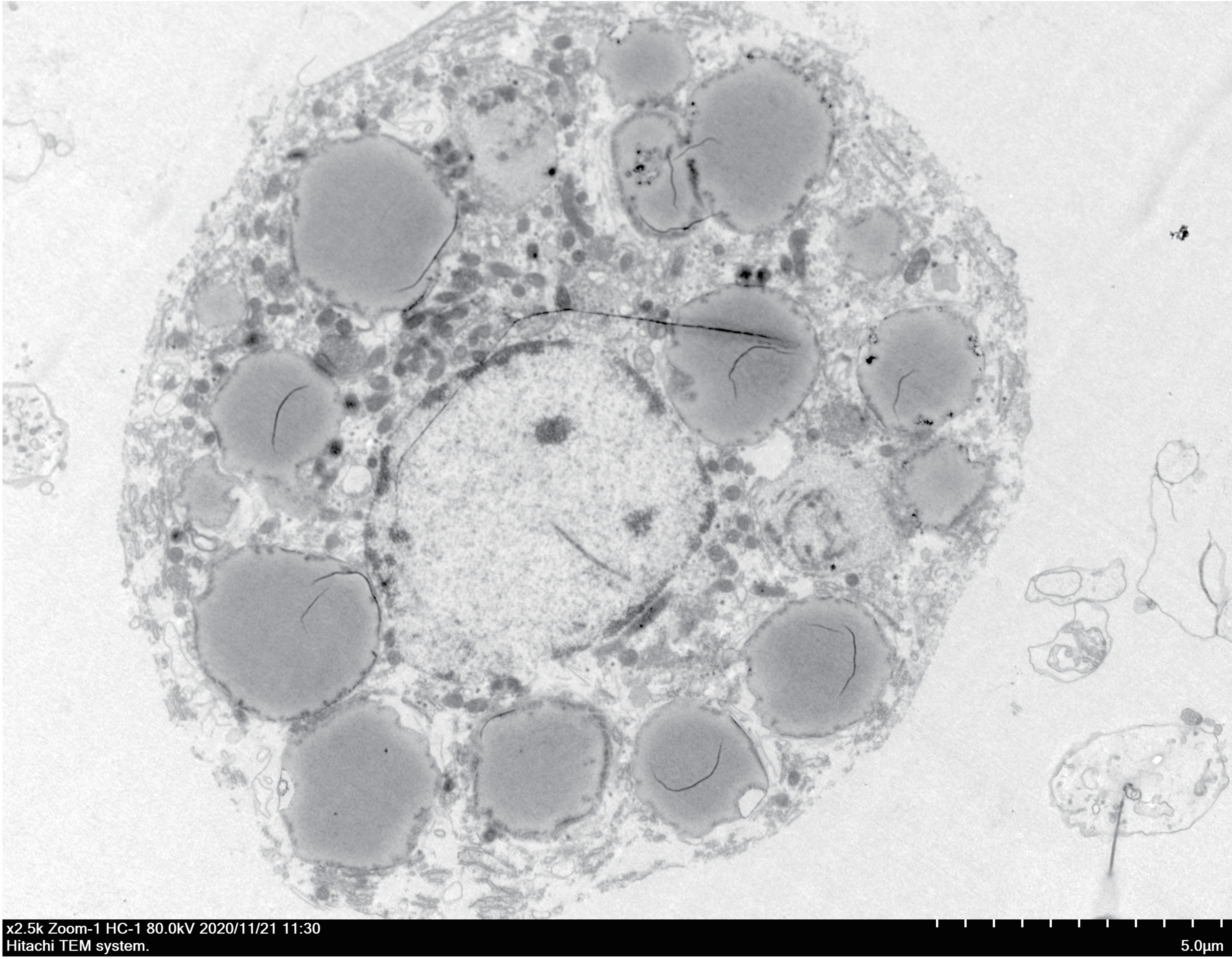

**Fig.4C**

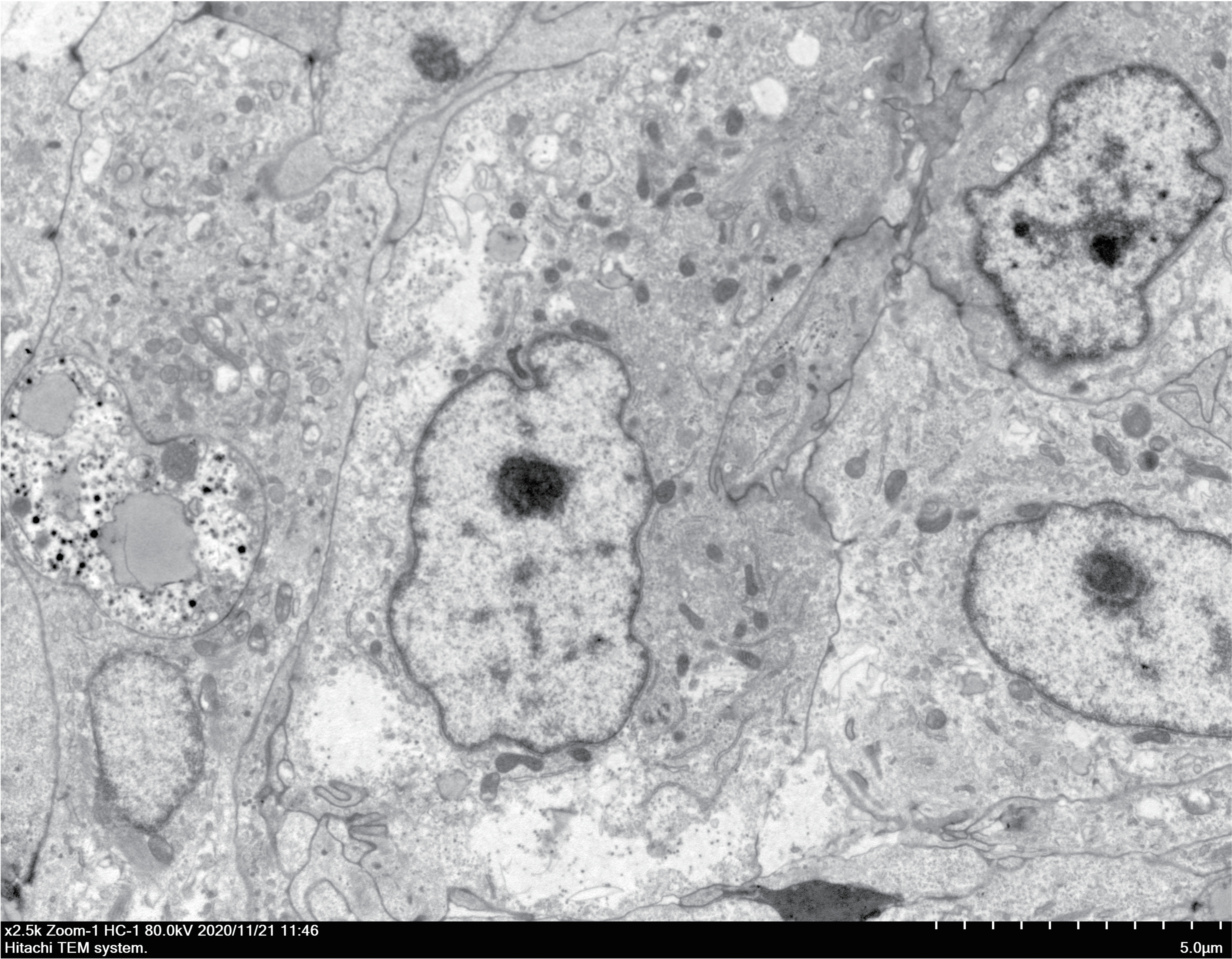

**Fig.4D**

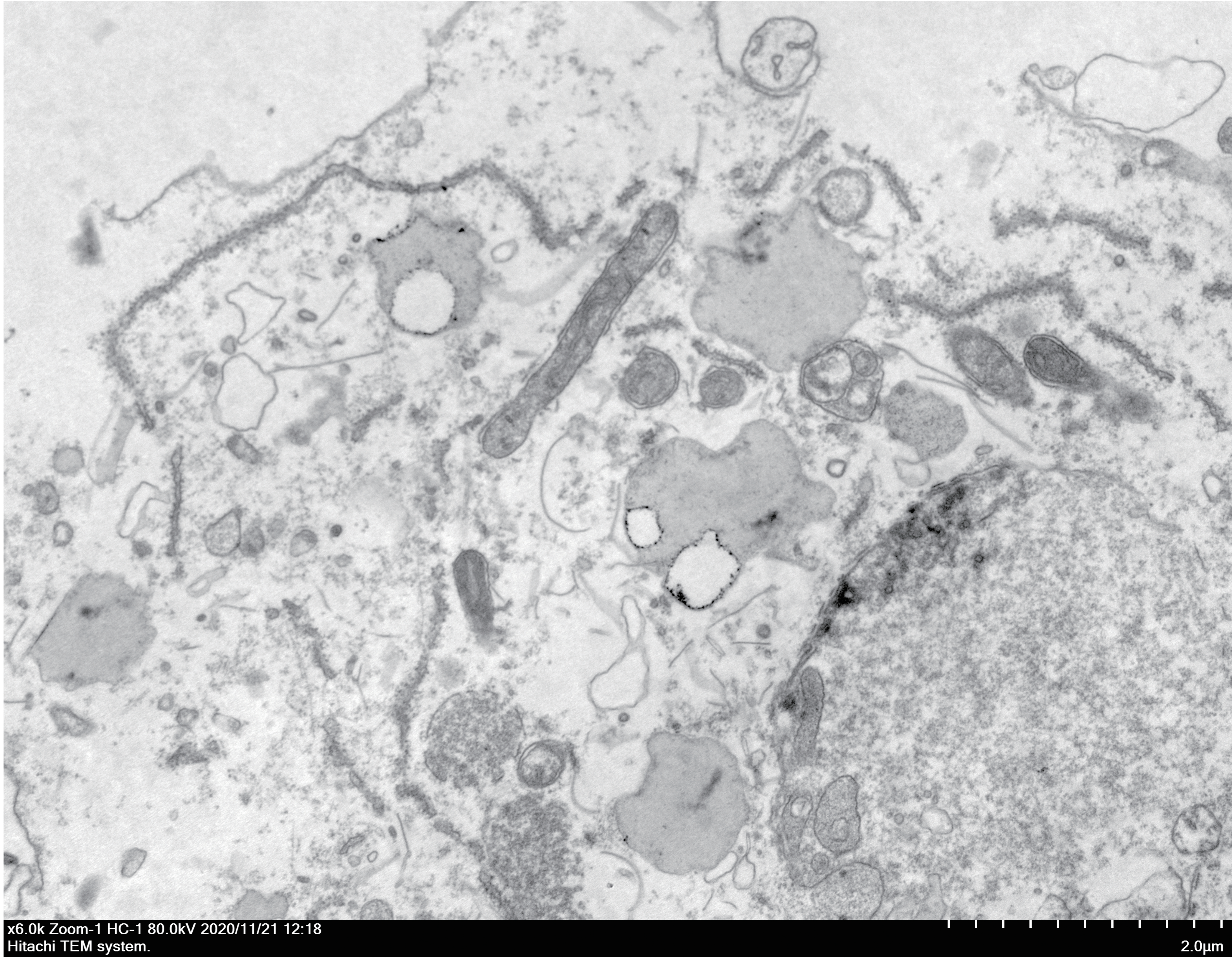

Supplement: S2 Raw images — (PDF) [file pone.0309976.s003.pdf]
